# *p*-Cresyl Sulfate

**DOI:** 10.3390/toxins9020052

**Published:** 2017-01-29

**Authors:** Tessa Gryp, Raymond Vanholder, Mario Vaneechoutte, Griet Glorieux

**Affiliations:** 1 Department of Internal Medicine, Nephrology Division, Ghent University Hospital, 9000 Ghent, Belgium; tessa.gryp@ugent.be (T.G.); raymond.vanholder@ugent.be (R.V.); 2 Laboratory for Bacteriology Research, Department of Clinical Chemistry, Microbiology & Immunology, Ghent University, 9000 Ghent, Belgium; mario.vaneechoutte@ugent.be

**Keywords:** *p*-cresyl sulfate, intestinal microbiota, chronic kidney disease

## Abstract

If chronic kidney disease (CKD) is associated with an impairment of kidney function, several uremic solutes are retained. Some of these exert toxic effects, which are called uremic toxins. *p*-Cresyl sulfate (pCS) is a prototype protein-bound uremic toxin to which many biological and biochemical (toxic) effects have been attributed. In addition, increased levels of pCS have been associated with worsening outcomes in CKD patients. pCS finds its origin in the intestine where gut bacteria metabolize aromatic amino acids, such as tyrosine and phenylalanine, leading to phenolic end products, of which pCS is one of the components. In this review we summarize the biological effects of pCS and its metabolic origin in the intestine. It appears that, according to in vitro studies, the intestinal bacteria generating phenolic compounds mainly belong to the families *Bacteroidaceae, Bifidobacteriaceae, Clostridiaceae, Enterobacteriaceae, Enterococcaceae, Eubacteriaceae, Fusobacteriaceae, Lachnospiraceae, Lactobacillaceae, Porphyromonadaceae, Staphylococcaceae, Ruminococcaceae*, and *Veillonellaceae*. Since pCS remains difficult to remove by dialysis, the gut microbiota could be a future target to decrease pCS levels and its toxicity, even at earlier stages of CKD, aiming at slowing down the progression of the disease and decreasing the cardiovascular burden.

## 1. Introduction

When kidney function deteriorates, a myriad of compounds, the so-called uremic retention solutes, accumulate in the circulation and tissues [1,2,3]. As a consequence, progression of kidney dysfunction is paralleled with the development of complications, affecting both the quality of life and survival of patients with chronic kidney disease (CKD) [4,5,6,7]. Uremic retention solutes are, conventionally, classified into three groups based on their physicochemical characteristics [2]. Next to the small water-soluble compounds (<500 Da) and the larger middle molecules (mostly peptidic compounds with molecular weight >500 Da) the protein-bound compounds form a third group. Many of these compounds, amongst which the phenols and indoles, originate from the intestinal microbial metabolism of dietary amino acids [8]. Whereas in the healthy kidney, removal of protein-bound solutes largely depends on tubular secretion, removal by dialysis, primarily replacing the filtration capacity of the kidney, is limited to the unbound fraction and is not affected by dialyzer pore size [9], and only marginally by convection [10]. Hence, dialysis removal is unsatisfactory, compared to, e.g., the low molecular weight compounds which are not bound to protein. For the protein-bound compounds alternative measures, preferably preventive (such as decreasing generation, adsorption, and preserving kidney function), will be necessary to keep concentrations low. In this review, *p*-cresyl sulfate (pCS), a prototype protein-bound molecule, to which many biological and biochemical (toxic) actions have been attributed, and for which increased concentrations have been associated with worsening outcomes (see below), will be discussed in depth. We will especially focus on the intestinal origin of pCS and the contributing bacterial species which could be a future target to decrease levels and hence toxicity, even at earlier stages of CKD, aiming at slowing down the progression of the disease and decreasing the cardiovascular burden.

## 2. Characteristics of *p*-Cresyl Sulfate

### 2.1. Chemical Structure and Molecular Weight

**Figure 1:**
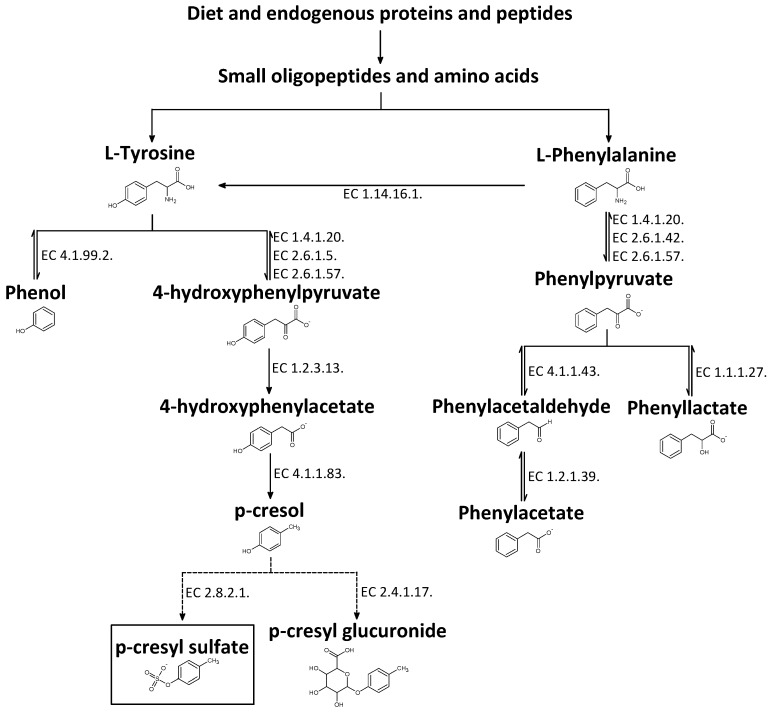
Conversion pathway of tyrosine and phenylalanine into *p*-cresyl sulfate. Full arrows: process through bacterial fermentation; dotted arrows: process through enzymatic reactions of the host; EC: enzyme commission number. l-tyrosine, derived from diet and endogenous proteins and peptides, can be converted to phenol and 4-hydroxyphenylpyruvate. Tyrosine phenol-lyase (EC 4.1.99.2.), previously named β–tyrosinase, is responsible for the reversible deamination of l-tyrosine, requiring pyridoxyl phosphate as a cofactor, into phenol ammonia and pyruvate [40,41,42]. This reaction is also reversible by the same enzyme using l-serine and phenol as substrates [43]. In addition, the reversible reaction of l-tyrosine with 2-oxoglutarate in 4-hydroxyphenylpyruvate and L-glutamate is catalysed by tyrosine transaminase (EC 2.6.1.5.) or by aromatic-amino-acid transaminase (EC 2.6.1.57.) [44,45,46]. To a small extent, 4-hydroxyphenylpyruvate and ammonia can also be formed by the enzyme phenylalanine dehydrogenase (EC 1.4.1.20.) from l-tyrosine [47]. 4-Hydroxyphenylpyruvate is the precursor of 4-hydroxyphenylacetate, catalysed by p-hydroxyphenylpyruvate oxidase (EC 1.2.3.13.) [44,46], and can subsequently lead to the formation of *p*-cresol by p-hydroxyphenylacetate decarboxylase (EC 4.1.1.83.) [48,49]. In the gut mucosa and in the liver, the majority of *p*-cresol will be conjugated into the uremic toxin *p*-cresyl sulfate by aryl sulfotransferases (EC 2.8.2.1.) [50] and a small fraction will be metabolized to *p*-cresyl glucuronide by UDP-glucuronyltransferases (EC 2.4.1.17.) [51]. Another aromatic amino acid, phenylalanine, also plays a role in the production of *p*-cresyl sulfate through the hydroxylation reaction to tyrosine by phenylalanine 4-monooxygenase, also referred as phenylalanine hydroxylase (EC 1.14.16.1.) [52]. This metabolic process is carried out by bacteria as well by liver cells, transforming excess diet phenylalanine to tyrosine [53]. In addition, phenylalanine is converted into 3-phenylpyruvate by either phenylalanine dehydrogenase (EC 1.4.1.20.) [47,54], branched-chain-amino-acid transaminase (EC 2.6.1.42.) [55] or by aromatic-amino-acid transaminase (EC 2.6.1.57.) [45]. Then 3-phenylpyruvate can be transformed in 3-phenylacetaldehyde by phenylpyruvate decarboxylase (EC 4.1.1.43.) [56] or in 3-phenyllactate by l-lactate dehydrogenase (EC 1.1.1.27.) [46]. Finally, 3-phenylacetaldehyde can be converted to 3-phenylacetate by phenylacetaldehyde dehydrogenase (EC 1.2.1.39.) [56,57]. All of these reactions of the phenylalanine metabolic pathway are reversible, which can lead, in the end, to *p*-cresyl sulfate generation.

The aromatic pCS originates from sulfation (para-) of the intestinally generated *p*-cresol (MW: 108.1 g/mol). pCS (C_7_H_8_O_4_S) (Figure 1) has a molecular weight of 188.2 g/mol and in the circulation (plasma) it is bound for approximately 95% to the protein albumin in healthy controls, as well as in CKD patients [11].

### 2.2. Normal and Uremic Serum/Plasma Concentrations

Normal total average serum/plasma concentrations of pCS reported, vary between 2.8 ± 1.7 mg/L (14.9 ± 9.0 µM) [12] and 6.6 ± 3.7 mg/L (35.1 ± 19.7 µM) [13] as determined in serum by ultra-performance liquid chromatography (UPLC) and UPLC-mass spectrometry (MS)-MS, respectively. In end-stage kidney disease (ESKD), concentrations of pCS are significantly increased with total average concentrations varying from 21.8 ± 12.4 mg/L (115.8 ± 65.9 µM) [14] to 106.9 ± 44.6 mg/L (568.0 ± 237.0 µM) [15], as quantified by UPLC in serum and LC-MS-MS in plasma, respectively.

## 3. Metabolism

### 3.1. p-Cresyl Sulfate, a Colon-Derived Solute

The gut microbiota plays an important role in human health and disease [16,17], with alterations of the intestinal microbiota linked to the development of different diseases, such as inflammatory bowel disease [18], cancer [19], obesity [20], diabetes [21,22], cardiovascular disease [23], and kidney disease [24]. Next to protection against invading pathogens and differentiation of the human immune system, the microbiota contributes to various metabolic functions, such as fermentation of non-digestible dietary compounds in the large intestine [17,25,26]. Approximately 6–18 g of proteins and peptides enters the large intestine every day, mostly from diet, and to a lesser extent from endogenous sources, such as host tissues, pancreatic enzymes, and other secretions [27,28,29]. Entering the large bowel, these substances undergo depolymerization by host- and bacteria-derived proteases and peptidases into small oligopeptides and amino acids. These small oligopeptides and amino acids are then available for assimilation by colon microbiota or can be further metabolized by host enzymes [30,31]. Predominantly in the distal part of the colon, the aromatic amino acids tyrosine and phenylalanine are converted into phenolic compounds, such as phenol and *p*-cresol, through a series of deamination, transamination, and decarboxylation reactions by bacterial fermentation [29,32] (Figure 1). Detoxification of phenols occurs in the mucosa of the colon [33] and in the liver [34], where, e.g., *p*-cresol is sulfated into pCS and a small fraction is glucuronated into *p*-cresyl glucuronide (pCG) [35,36]. When entering the circulation these solutes bind to plasma albumin in a reversible manner, such that a rapid equilibrium between the bound and free fraction is established [37]. Finally, under normal conditions, the free fraction of these compounds is filtered at the site of the glomerulus and the bound fraction is secreted at the site of the tubular epithelial cells and both end up in the urine. In CKD patients, excretion of these solutes is impaired, resulting in their accumulation. Of note, in CKD estimates of proximal tubular secretion function and glomerular filtration of pCS correlate to each other to a certain extent, but that substantial variability in the change of net secretion vs. normal by the two components of the kidney remains [38]. In many studies plasma/serum and urine levels of *p*-cresol are reported. It is of note that these levels reflect the sum of both conjugates, pCS and pCG, since *p*-cresol is not present at detectable levels in the circulation [39].

The degradation routes by the gut microbiota of the aromatic amino acids tyrosine and phenylalanine are known to a large extent. As shown in Figure 1, several phenolic compounds and intermediates are generated from tyrosine and phenylalanine through intestinal bacterial fermentation [40,41,42,43,44,45,46,47,48,49,50,51,52,53,54,55,56,57].

Smith et al. demonstrated that, in the large intestine, bacterial fermentation of proteins mainly occurs in the distal part of the colon, since they found a four-fold higher concentration of phenolic compounds in that section of the intestine compared to the proximal bowel. In the distal colon, phenol and *p*-cresol account for 70% of all products of the dissimilatory aromatic amino acid metabolism [29]. Accordingly, in batch culture incubations with human faecal slurries, phenol and *p*-cresol are the predominant end products of tyrosine fermentation [29,32], whereas the predominant end products of phenylalanine are phenylacetate and phenylpropionate [29].

### 3.2. Disturbed Protein Assimilation in CKD

Assimilation of protein (digestion, metabolism, and absorption) in the small intestine is impaired in both non-dialyzed and dialyzed CKD patients [58,59] which might contribute to protein malnutrition, a well-known problem in CKD patients [60,61]. The ^13^C protein breath test is used to measure the impairment of protein assimilation in CKD, which worsens as kidney function declines. Proteins which are not absorbed or digested in the small intestine are further metabolized in the colon, by the gut proteolytic bacteria, which will cause an increase of urinary *p*-cresol concentration [58,59], an indicator of colonic *p*-cresol generation [62,63]. In the small intestine, protein absorption and digestion are also dependent on the degree of dietary protein intake, which is decreased in many malnourished uremic patients. This should have a lowering effect on urinary *p*-cresol [62,64]. However, in the study of Bammens et al., urinary *p*-cresol levels were still more pronounced as kidney function declined in CKD patients, after normalisation for dietary protein intake [58]. Gastro-intestinal motility disorders and dysbiosis in the gut should also be taken into account when evaluating protein assimilation [58,59]. Conditions that can interfere with protein assimilation are hepatic failure [65], diabetes mellitus [66], and metabolic acidosis [67]. Whereas hepatic failure and especially diabetes mellitus are frequent causes of CKD, acidosis is a frequent complication in uremia, accelerating amino acid and protein catabolism by, e.g., increasing muscle protein breakdown [67,68]. Correction of metabolic acidosis in CKD patients results in a normalization of the catabolic response to a low-protein diet by reducing amino acid oxidation and protein degradation, and stimulating protein synthesis to normal levels [68].

## 4. Gut Microbiota

### 4.1. Intestinal Phenolic Compounds Generating Microbiota

In general, the most abundant bacterial phyla among the human adult gut microbiota are Firmicutes, Bacteroidetes, Actinobacteria, Proteobacteria, and Verrucomicrobia and, to a lesser extent, Cyanobacteria, Fusobacteria, Lentisphaerae, Spirochaetes, and TM7 [69]. Obligate anaerobic bacterial genera such as *Bacteroides*, *Bifidobacterium*, and *Eubacterium* predominantly inhabit the human colon, while bacterial species of the genera *Clostridium*, *Enterococcus*, and *Lactobacillus* are present to a lesser extent [70,71]. Macfarlane et al., found in 1986 that proteolytic bacterial species in faeces are predominantly *Bacteroides* spp. and *Propionibacterium* spp. and belong, in a lesser proportion, to the genera *Bacillus*, *Clostridium*, *Staphylococcus*, and *Streptococcus* [30]. Gut microbiota are known to be the source of phenolic compounds, generated in the colon from tyrosine and phenylalanine, as demonstrated in hemodialysis (HD) patients without colon who have the same plasma pCS levels as healthy subjects [72]. This was also demonstrated by Wikoff et al., who could not detect pCS in plasma of germ-free mice [73] and by Kikuchi et al., who observed a non-significant downward trend in urinary pCS levels in cecectomized rats [74]. Two operational taxonomic units (OTUs), belonging to the genus *Oscillospira* and the family *Ruminococcaceae*, are associated with urinary pCS levels and one OTU, belonging to the family *Ruminococcaceae*, is associated with levels of pCG in healthy subjects [75]. In vitro studies have been carried out to identify the microbiota generating phenolic compounds. Table 1 gives an overview of culturable phenolic compound generating bacteria with predominant phenol-producing bacteria belonging to the *Enterococcaceae*, *Clostridiaceae*, *Staphylococcaceae*, and *Enterobacteriaceae*, of which the latter group is known to produce the enzyme tyrosine phenol-lyase [40,43]. Most studied proteolytic bacteria are of the genus *Clostridium* [76,77,78,79], of which some can use amino acids as their sole carbon and energy source [76,77]. Additionally, *Bifidobacterium* strains are known to ferment aromatic amino acids in the absence of other energy sources such as carbohydrates [70]. Next to phenol, the main tyrosine end products of several *Clostridium* species are *p*-cresol and 4-hydroxyphenylacetate, the precursor of *p*-cresol. Additionally, *Bacteroidaceae*, *Bifidobacteriaceae*, *Eubacteriaceae*, *Lachnospiraceae*, *Porphyromonadaceae*, *Ruminococcaceae*, and *Veillonellaceae* have the property to ferment tyrosine to *p*-cresol and to 4-hydroxyphenylacetate, while *Fusobacteriaceae* only generate *p*-cresol (Table 1). Bone et al. hypothesized that in the large intestine, phenol-forming bacteria are mainly facultative anaerobes, while *p*-cresol-forming bacteria are obligate anaerobes [80]. This hypothesis largely corresponds to the data presented in Table 1. The kind of phenolic compounds that are generated by gut bacteria can depend on the bacterial strain under study, but also largely on the carbon and energy source, whereby in vitro study of external factors, such as medium composition and incubation conditions, can have a large impact on protein dissimilation [70]. Since only a small fraction of the bacterial gut community is culturable [81], it can be suspected that the aromatic amino acid fermenting bacteria listed are only the tip of the iceberg. For future directions, culture conditions will have to be optimized in order to increase the identification yield of these phenolic compound generating bacteria.

### 4.2. Gut Dysbiosis in CKD

It has been shown that the composition of the gastro-intestinal microbiota of CKD patients is altered [82]. More specifically, CKD patients suffer from bacterial overgrowth in the small intestine [83] with elevated levels of aerobic (10^6^ cells/mL) and anaerobic (10^7^ cells/mL) bacteria in the duodenum and jejunum [84] and overgrowth of aerobic bacteria in the colon [85,86]. Alterations in the gut microbiota composition are also observed in nephrectomized rats, who have an increase of *Bacteroides* species and a decrease of *Lactobacillus* species compared with sham-operated rats [87]. Changes in the gut bacterial species composition may even begin in early stages of CKD, as increased levels of pCS are associated with an increase of an OTU belonging to the *Ruminococcus* genus in the early stages of CKD [88]. Vaziri et al. demonstrated that the relative abundance of 190 bacterial OTUs differed between controls and ESKD patients, whereby ESKD patients had higher amounts of aerobic and facultative anaerobic bacteria, with the largest increases in the Clostridia, Actinobacteria, and Gammaproteobacteria [82]. To distinguish the effect of uremia, per se, from inter-individual variations, Vaziri et al. investigated the gut microbial composition in nephrectomized rats. Differences in 175 bacterial OTUs were found with, more specifically, a decrease in the *Lactobacillaceae* and *Prevotellaceae* families in uremic rats. The total richness was significantly higher in control rats than in the nephrectomized rats [82]. Additionally, in HD patients, analysis of the faecal microbial composition by culturing techniques revealed an approximately 100 times higher abundance of aerobes, such as *Enterobacteriaceae* and *Enterococcaceae*, compared to healthy individuals. Of the anaerobes in HD patients, bifidobacteria were decreased while the number of *Clostridium perfringens* increased [85]. These findings are consistent with the results of Fukuuchi et al., studying the faecal microbial composition by traditional plating methods in CKD, HD, and control patients. However in this study, the total amount of bacteria was decreased in the HD and CKD groups [86], while Hida et al. found no differences [85]. Among the *Enterobacteriaceae*, the level of *Escherichia coli* was significantly increased in both patient groups compared to the control group [86]. However, when using real-time polymerase chain reaction (PCR) analysis on faecal samples, no difference was observed in the *E. coli* level between peritoneal dialysis (PD) patients and controls, while the *Bifidobacterium* species decreased [89]. According to Table 1, these studies indicate a higher prevalence of phenol-producing bacteria belonging to the *Enterobacteriaceae* and *Enterococcaceae* families and of *p*-cresol-producing bacteria, like *C. perfringens* in CKD patients. This has been confirmed by the quantification of the faecal and serum levels of phenol and *p*-cresol, which are higher in HD and CKD patients than in controls [85,86]. The lowered levels of bifidobacteria in CKD, HD and PD [85,86,89] indicate that these bacterial species are probably not responsible for the increased production of phenol and *p*-cresol in these patients, although bifidobacteria have been found to generate phenolic compounds in in vitro studies (Table 1). From the section above, it becomes clear that the information in this field of research is still scattered and incomplete, underscoring that the characterization of the uremic intestinal microbiota will require further investigation before the composition of the responsible bacterial species, contributing to the increased pCS and phenyl sulfate levels in CKD patients has been unraveled completely.

Next to the gut microbiota composition, also its functional characteristics play a role in determining the gut levels of *p*-cresol. The altered composition of the gastro-intestinal microbiota in CKD [82] is probably due to bacteria-derived hydrolysis of increased concentrations of urea entering the intestine via the entero-hepatic cycle, resulting in high levels of ammonia, which elevates the faecal pH value [90]. Wong et al. demonstrated that 19 microbial families were dominant in ESKD compared to controls, and that 63% of these dominant microbial families possessed urease-forming enzymes, needed to convert urea in ammonia. Other dominant families in ESKD (i.e., *Clostridiaceae* and *Enterobacteriaceae*) possess uricase- and *p*-cresol-forming enzymes, while a lowered family abundance was found for *Lactobacillaceae* and *Prevotellaceae*, producing butyrate-forming enzymes [91] which can further influence the butyrate production, knowing that this short chain fatty acid has a beneficial effect on the gastro-intestinal health [69]. Thus, uremia can modify the biochemical milieu of the gastro-intestinal tract. In addition to uremia itself, therapeutic interventions and dietary restrictions will also contribute to an altered gut microbiota composition and function [82].

## 5. Toxicity

### 5.1. Biological Effects

Literature reports on biological activity of pCS only emerged during the last decade, as until 2005, due to a preparative artefact caused by using acidification for deproteinization of plasma, its intestinally-generated precursor, *p*-cresol, was thought to be retained in the circulation [39]. Shortly after de Loor et al. [36] and Martinez et al. [103] reported this phenomenon independently from each other by using alternative deproteinization protocols (acetone and methanol, respectively), a first in vitro study by Schepers et al. pointed to a role of pCS in causing increased oxidative stress in leukocytes [104]. In vivo intravital microscopy in rat peritoneal capillary venules confirmed the stimulatory effect of pCS on leukocytes showing an increase in the number of rolling leukocytes along the vascular endothelium after superfusion of the peritoneal membrane with a solution containing pCS at uremic concentration [105]. Furthermore, pCS stimulated endothelial microparticle release, a marker of endothelial damage [106], and induced oxidative stress in both human umbilical vein endothelial cells (HUVECs) and human vascular smooth muscle cells (HVSMCs) [107]. Ex vivo, pCS induced contraction of mouse thoracic aorta, through direct activation of rho-kinase, independently of oxidative stress induction, as well as inward eutrophic vascular remodelling [107]. Furthermore, pCS induced NADPH oxidase activity and reactive oxygen species (ROS) production in cardiomyocytes facilitating cardiac apoptosis and resulting in diastolic dysfunction in nephrectomized mice [108]. These data suggest that pCS might contribute to cardiovascular morbidity and mortality in CKD.

In addition, toxic effects on renal tubular cells have also been reported. pCS increased expression of DNA methyltransferases 1, 3a, and 3b isoforms which suppressed Klotho expression in HK2 cells and injection of pCS in uninephrectomized B-6 mice caused kidney fibrosis, CpG hypermethylation of the klotho gene, and decreased klotho expression in the renal tubular cells [109]. Sun et al. also demonstrated that pCS activated the renal renin angiotensin aldosterone system/transforming growth factor-beta pathway and induced epithelial-to-mesenchymal transition-like transition contributing to kidney injury and fibrosis [110]. In parallel to what is observed at the cardiovascular level, pCS also induces NADPH oxidase driven production of ROS next to expression of inflammatory cytokines in renal tubular cells, a process involved in kidney fibrosis [111]. This pro-inflammatory, but also pro-apoptotic, effect of pCS on human proximal tubular epithelial cells (PTEC) was confirmed by Poveda et al. [112]. Interestingly, pCS was shown to inhibit the activity of the human conditionally immortalized PTEC efflux transporters Multidrug Resistance Protein 4 (MRP4) and Breast Cancer Resistance Protein (BCRP), by 40% and 25%, respectively. These are two efflux transporters involved in pumping solutes out of the tubular cell, of which inhibition may possibly lead to intracellular accumulation and increased toxicity of the substrates of those transporters which are various organic acids amongst which pCS, which may contribute to progression of CKD [113].

Finally, pCS was shown to play a role in insulin resistance, in aberrant adipose tissue metabolism, and reallocation of fat in the body [114], in hampering calcium deposition and osteoprotegrin expression in human mesenchymal stem cells and in suppressing the immune response by T_h_1-cells [115] and macrophages [116].

Hence, pCS contributes to many mechanisms that are involved in cardiovascular and renal damage. The question is whether these experimental findings are corroborated in clinical studies.

### 5.2. Clinical Associations

As summarized in Table 2, both increasing levels of total and free pCS [117,118,119,120,121,122,123,124,125,126,127,128], as well as its urinary excretion [129], have been repeatedly associated with cardiovascular complications and mortality in patients with CKD whether or not on dialysis [130]. In addition, total pCS has been linked to progression of renal failure [131]. Recently, total pCS also has been associated with pruritus in CKD [132].

## 6. Therapeutic Methods for Reducing *p*-Cresyl Sulfate Concentration

### 6.1. Affecting the Generation of pCS

Most strategies to lower plasma pCS levels (summarized in Table 3) are based on solute removal by dialysis (see below) and are, to a lesser extent, focused on suppressing solute production.

#### 6.1.1. Diet

Diet has a major effect on the gut microbial composition, considering that healthy colon microbiota is primarily composed by saccharolytic bacteria whereas, in CKD, proteolytic bacteria predominate. Saccharolytic bacterial species mainly ferment carbohydrates, which yields short chain fatty acids, such as acetate, butyrate, and propionate, which are beneficial to the host [17]. Proteolytic bacterial species, on the other hand, predominantly metabolize proteins resulting in the generation of a variety of end products including short or branched chain fatty acids, ammonia, amines, thiols, phenols, and indoles, some of which have toxic properties [134]. Thus, a potential way to lower the generation of proteolysis-derived microbial metabolites such as phenol and *p*-cresol is trying to alter the gut metabolism in favour of a saccharolytic profile by increasing the dietary intake of complex carbohydrates and fibers while decreasing dietary protein intake [29,134], and some data indeed suggest that this is the case [17,135]. Exclusion of dietary protein intake by adhering to a vegan diet was shown to decrease bacterial urease activity by 66% [136], and also to cause a decline of serum and urinary *p*-cresol and phenol levels [136]. Patel et al. demonstrated a decrease of urinary pCS levels by 62% in healthy vegetarians, who consume 25% less protein and 69% more fibre than individuals consuming an unrestricted diet [137]. To the contrary, when protein intake was enriched by 8.4% in healthy humans, urinary *p*-cresol levels increased from a mean concentration of 7.71 ± 2.47 mg/g creatinine in the control period to 17.59 ± 4.58 mg/g creatinine in the protein rich period [64]. This is in agreement with a study in healthy subjects on a controlled diet, whereby increasing the protein intake by 73.3 g/day, mainly by increasing meat intake, resulted in a an elevation of the level of total urinary volatile phenol from a concentration of 74 ± 14.5 mg/day in the low protein diet period to 108 ± 14.6 mg/day in the high protein diet period. On other hand, addition of 29.8 g of wheat fibre per day to the high protein diet induced a non-significant downward trend in total urinary phenol levels [138]. However, for some bacterial species the presence of carbohydrates also stimulates the dissimilation of aromatic amino acids [29] or is needed for activating the aromatic amino acid metabolism because of their inability to use amino acids as their sole carbon and energy source [93]. Birkett et al. demonstrated in healthy volunteers that a diet consisting of high resistant starch decreased faecal *p*-cresol and phenol concentrations by about 29 ± 2 µg/g and 1.0 ± 0.1 µg/g, respectively, however without a significant effect on urinary *p*-cresol concentrations [139]. Rossi et al. showed that carbohydrates are an important factor in reducing free and total pCS levels in non-dialyzed CKD patients, with total dietary fibre, negatively, and protein-fibre index, positively associated with total serum pCS [140]. Additionally, in HD patients, increased dietary fibre intake resulted in a lower free plasma pCS [141]. An animal study in uremic rats ingesting fermentable dietary fibre high amylose maize resistant starch type 2 (HAMRS2) resulted in a decrease of cecal pH and urinary *p*-cresol by 47%. The microbial diversity was also decreased in this group while the Bacteroidetes-to-Firmicutes ratio increased. The latter is considered to be an indicator of a healthy gut [142]. It is of note a rigid dietary protein restriction in CKD/HD patients is not a good solution to diminish uremic toxins level because inadequate dietary protein intake contributes to malnutrition [58]. According to the British diabetic association evidence-based guidelines, a minimum protein intake of 1.1 g/kg ideal body weight (IBW)/day and 1.0–1.2 g/kg IBW/day is required for HD and PD patients, respectively [143]. Irrespective of protein intake, patients with CKD are in addition often prescribed a combined diet to prevent fluid overload, hyperphosphatemia and hyperkalemia, with restrictive intake of fruits, vegetables, and high-fibre products [82], which is likely to alter the fermentation in favour of a proteolytic profile, increasing the generation of the unwanted phenolic compounds. In a meta-analysis, supplementation of a restricted protein diet with ketoacid analogues of essential amino acids has been suggested to delay the progression of CKD effectively without causing malnutrition [144]. In addition, this therapy has been shown to improve the native arteriovenous fistula maturation, to decrease the initial vascular stiffness and to limit inflammatory response in CKD patients [145]. However, the effects of keto-analogues on intestinal microbiota and on levels of intestinally-generated uremic toxins has to our knowledge as yet not been evaluated.

#### 6.1.2. Probiotics, Prebiotics, and Synbiotics

Several studies demonstrated favourable effects on *p*-cresol metabolism due to the administration of probiotics, prebiotics, and synbiotics [85,146,147,148,149,150,151,152,153,154,155,156,157]. In general, pre- and probiotics aim to increase the saccharolytic activity of colonic bacteria because of the beneficial effects attributed to the end products of carbohydrate fermentation and to decrease the generation of the proteolytic fermentation end products of which some have toxic effects [146]. Administration of the probiotic *Lactobacillus acidophilus* (1 × 10^10^ CFU/kg/day) mitigated urinary protein excretion in nephrectomised rats and lowered serum pCS levels. However, administration of *L. acidophilus* had no effect on faecal *p*-cresol and phenol levels. This suggests that the reduced serum pCS levels are primarly due to blocking of *p*-cresol entry from the intestine into the circulation through restoring the intestinal tight junction protein expression by *L. acidophilus* [87]. With regard to the effect of probiotics on the pCS levels in healthy humans, as reviewed by Rossi et al. [147], it has been shown that probiotic *Lactobacillus* strains are associated with a decreased urinary *p*-cresol excretion [148] along with decreased faecal bacterial β-glucuronidase, nitroreductase and glycocholic acid hydrolase enzyme activities [158]. At least one strain of *Lactobacillus gasseri* has the ability to decrease faecal *p*-cresol levels in healthy subjects and to lower the number of cells of *Staphylococcus* [149], a proteolytic bacterium [30]. Also dietary addition of the probiotic *Lactobacillus casei Shirota* strain or the probiotic *Bifidobacterium breve Yakult* strain resulted in a decrease of urinary *p*-cresol in healthy volunteers [146]. Furthermore, Lebenin, an oral preparation of lactic acid bacteria containing *Bifidobacterium infantis*, *Enterococcus faecalis*, and *L. acidophilus* decreased faecal *p*-cresol levels in healthy persons [85]. In contrast, in pediatric HD and PD patients, a high concentration of a probiotic preparation, containing *Lactobacillus* spp., *Bifidobacterium* spp., and *Streptococcus salivarius* subsp. *thermophilus*, had no effect on the serum pCS levels [159]. Lowering the pH value by lactic acid producing bacteria, such as *Lactobacillus* and *Streptococcus* spp., reduced the dissimilatory metabolism of aromatic amino acids [29] and suppressed aerobic bacterial overgrowth [85], which in its turn reduces the accumulation of pCS.

Administration of prebiotics, such as lactulose or oligofructose-enriched inulin (OF-IN) to healthy humans, resulted in a significant reduction of *p*-cresol in the urine for both substrates, with OF-IN having the largest effect (from 27.7 ± 15.3 mg/day to 17.8 ± 10.8 mg/day) compared to the lactulose group (from 20.7 ± 11.6 mg/day to 12.7 ± 8.9 mg/day) [150]. Using real-time PCR, it was shown that the total number of bifidobacteria in faeces were elevated after administration of both prebiotics [146,150]. Davis et al. demonstrated that administration of the prebiotic galacto-oligosaccharide (GOS) during 12 weeks to healthy humans resulted in a dose-dependent increased abundance of actinobacteria with specific enrichment of bifidobacteria at the expense of the Bacteroides group [151]. In HD patients, OF-IN decreased the pCS generation rate and serum pCS concentrations by 20% [152]. Acarbose, a small intestinal α-glucosidase inhibitor, is another prebiotic that is administered to increase undigested carbohydrate levels in the colon. Acarbose was shown to lower serum *p*-cresol concentration and urinary *p*-cresol excretion, the latter reflecting its colonic generation rate [153]. However, Poesen et al. could not establish any effect of the prebiotic arabinoxylan oligosaccharide on serum and 24 h urinary excretion pCS and pCG levels in CKD patients [154].

Finally, the combination of pre- and probiotics, the so-called synbiotics, more recently gained interest. In healthy humans, long-term administration of the *L. casei Shirota* strain in association with OF-IN resulted in a significant urinary *p*-cresol reduction [146]. Similar effects were shown in HD patients, in who synbiotic administration, consisting of the *L. casei Shirota* strain, the *B. breve Yakult* strain, and GOS, decreased serum *p*-cresol levels [155]. Additionally, Probinul-neutro, a synbiotic containing *Lactobacillaceae*, *Bifidobactericeae*, *S. thermophilus*, inulin, and tapioca-resistant starch, reduced total plasma *p*-cresol concentrations in non-dialyzed CKD patient stages 3–4 [156]. In a randomized controlled trial (RCT), Rossi et al. recently demonstrated a significant reduction of serum pCS in CKD patients administered a combination of inulin, fructo-oligosaccharides, GOS, and different strains belonging to the genera *Lactobacillus*, *Bifidobacteria*, and *Streptococcus*. This decrease was even more marked if the patients who had received antibiotics in this study (about 1/3 of the enrolled subjects) were excluded [157].

#### 6.1.3. Laxatives

In CKD patients, the colonic transit time is prolonged mainly in the colon ascendens and in the recto-sigmoid segment [160]. Constipation was documented to occur more frequently in HD patients (63.1% of 268 patients) compared to continuous ambulatory peritoneal dialysis (CAPD) patients (28.9% of 204 patients) [161]. The higher frequency of constipation, in general, is due to several factors, such as dietary restrictions, medication, lifestyle, low fluid intake, and comorbidity [8]. Longer colonic transit time results in an elevated bacterial fermentation of amino acids [75,138] and may subsequently induce an overgrowth of proteolytic bacteria in the human colon. In a study of Roager et al., urinary pCS and pCG correlated positively with colonic transit time [75]. A consecutive stepwise three-stage in vitro culture model mimicking the colon with fresh faecal slurries as initial substrate entered into the system showed a higher *p*-cresol and phenol concentration with longer transit time [29]. From this point of view it might be interesting to study in future whether administration of laxatives might decrease pCS levels.

### 6.2. Adsorption

#### 6.2.1. AST-120

Decreasing the uptake of colon-derived solutes by administering oral adsorbents, such as AST-120, might be another option to lower pCS levels. Administration of AST-120 in CKD rats resulted in a decrease of serum pCS [162] and of *p*-cresol levels and a reduction of *p*-cresol urinary excretion [163]. This is in agreement with a recent study of Velenosi et al., demonstrating a significant reduction of pCS and pCG in plasma, heart, kidney, and liver of uremic rats after AST-120 administration [164]. Next to decreasing serum and urinary pCS levels, AST-120 also changed overall gut microbiota composition in uremic rats [74]. In dialyzed patients, treatment with AST-120 resulted in a decrease of total and free pCS plasma levels [165,166]. Thus, AST-120 is a potential treatment to lower pCS and even pCG levels in CKD patients, but this treatment does not have a lasting effect after intake discontinuation [165]. Two recent RCTs in CKD patients, in which plasma levels of pCS were not reported, showed no beneficial effect of AST-120 on the progression of CKD [167,168]. In one of these studies, however, the evolution of the concentration of another protein-bound compound, indoxyl sulfate (IxS), was followed, without changing a change in concentration in the group on AST-120 [168].

### 6.3. Preserving Kidney Function

In healthy kidneys, urinary excretion of protein-bound metabolites like pCS is, in large part, determined by tubular secretion rather than filtration, which shifts the binding and allows active secretion of these solutes. To mediate urinary solute excretion, renal proximal tubules are equipped with a range of transporters that cooperate in basolateral uptake (e.g., organic anion transporter (OAT)-1 and OAT-3) and luminal excretion, such as multidrug resistance protein (MRP)-4 and breast cancer resistance protein (BCRP). It is conceivable that these functions are lost as kidney failure progresses and that any intervention to prevent this progression will also result in less retention of uremic solutes, including pCS.

### 6.4. Dialysis and Renal Transplantation

#### 6.4.1. Dialysis

Due to their binding, protein-bound uremic toxins, such as pCS, are poorly filtered across dialysis membranes. Pre-and post-dilution hemodiafiltration (HDF) increased the reduction ratios of pCS when compared to high flux hemodialysis (HFHD) [169] and when compared to pre-dilution hemofiltration [170]. Thus, combining convective with diffusive removal seems to improve removal of protein-bound toxins during dialysis. However, these results were not confirmed in a study by Krieter et al. [171], who did not find superior reduction ratios for pCS with post-dilution HDF compared to HFHD. In a longitudinal setting, after six months of treatment with pre-dilution hemofiltration, the concentrations of total and free *p*-cresol, representing the combination of pCS and pCG, were reduced compared to low-flux dialysis used before the start of the study [10]. Similarly, a decrease in the predialysis concentration of total pCS was observed after nine weeks of post-dilution HDF compared to high-flux dialysis before the start of the study [172]. Even if reductions with convective strategies are significant, the question is whether these changes are clinically relevant. Increase in dialysate flow (QD) and dialyzer surface (KoA) is another way to increase the clearance of pCS [173]. The same group pointed to the fact that in dialysis patients, a notable fraction of the weekly solute removal of protein-bound toxins is accomplished by residual renal function (RRF) [174].

A possible promising strategy to optimize the removal of protein-bound toxins could be the use of sorbent technology in dialysis. Using fractional plasma separation and absorption (FPAD), the reduction ratios of *p*-cresol were doubled in comparison to HFHD. This study however, was discontinued due to serious clotting problems [175]. In a more recent study, removal rates of FPAD treatment in comparison to HFHD were 127% for *p*-cresol [176]. In an in vitro setting, pCS was fully absorbed from dialysate by a commercially available activated carbon sorber [177]. In pilot in vitro experiments, mixed matrix membranes, that contained incorporated activated carbon, adsorbed on average 2.27 mg pCS/g membrane in 4 h in diffusion experiments and 2.68 mg pCS/g membrane in convection experiments. It was estimated that the membranes would suffice to remove the daily production of pCS [178]. Recently, alternative sorbents were proposed. A nanoporous activated carbon monolith prototype designed for direct blood contact was shown to almost completely remove pCS in vitro [179] and highly porous microparticles prepared from poly(etherimide) were also able to highly absorb pCS with high affinity [180]. In an in vitro hemodiafiltration setting aiming to interfere with protein binding by increasing plasma ion strength (IPIS; hypertonic NaCl solution), the clearance of pCS was increased by 53.6% ± 10.2% [181]. In a pilot RCT comparing HDF-IPIS to HD and HDF only an increase in dialytic clearance of free IxS, but not of pCS was observed [182].

In PD, the total clearance, dialysis plus RRF, of *p*-cresol as surrogate for pCS was markedly lower compared to HFHD, in spite of less removal per unit of time with PD [183]. In contrast to the water-soluble compounds, PD clearance of pCS did not increase when RRF was lost, but nevertheless no rise in their plasma concentration occurred [184]. In a more recent prospective observational cohort of incident PD patients, PD clearance tended to increase, but did not compensate for the declining renal clearance, with as a consequence that serum pCS concentrations increased in parallel with loss of RRF [185]. The importance of RRF in removal of pCS in PD patients was recently confirmed by Huang et al. [186]. The fact that, despite the lower removal rate, the plasma concentration of protein-bound compounds is lower in PD patients that in HD patients [183,184], is suggesting that other, possibly metabolic, factors are determining pCS concentrations [187]. Additionally, in HD patients, concentrations of pCS seem dependent on protein equivalent of nitrogen appearance and not on dialysis adequacy as assessed by Kt/V (urea) [188].

#### 6.4.2. Renal Transplantation

Finally, with renal transplantation, decreases of serum pCS (and pCG) were observed at each of the analysed time points post-transplantation (day 7 and three and 12 months) [189]. Levels were significantly lower in transplant recipients when compared with CKD control patients with the same kidney function. Further analysis demonstrated significantly lower 24 h urinary excretion of these solutes in transplant recipients in spite of virtually identical glomerular filtration rates (GFR) [190]. Thus, differences in GFR do not seem to play a major role in this lower concentration of pCS among renal transplants. These changes may be due to immunosuppressive agents, antibiotics, other drugs, the transplantation procedure itself [191].

## 7. Conclusions and Future Perspectives

The protein-bound uremic toxin, *p*-cresyl sulfate, originates from the bacterial metabolism in the intestine and several phenolic compound-generating bacteria have been identified. Circulating levels of pCS are increased in CKD and increased levels have been associated with worse outcome of CKD patients. The currently available therapeutic methods trying to decrease levels of pCS, among other protein-bound uremic toxins, still remain inadequate. Targeting its generation seems an attractive measure, which could not only be applied as a preventive measure at earlier stages of CKD, but also in addition to dialysis at the end stage. Development of intervention strategies at the level of the intestinal microbiota first needs a thorough characterization of the uremic microbiome and its functional capacity.

## Figures and Tables

**Table 1:** Overview of the phenolic compound generating bacterial species (in vitro literature data).

Bacterial Species	Tyrosine End Products			Phenylalanine End Products	
	Phenol	*p*-Cresol	4-Hydroxy-Phenyl-Acetate	Phenyl-Acetate	Phenyl-Lactate
**FIRMICUTES**					
Clostridiaceae					
*Clostridium aminovalericum*				[92]	
*Clostridium bartletti*		[93]	[93]	[93]	[93]
*Clostridium bifermentans*	[29]			[76,92]	[29,76]
*Clostridium botulinum type G*			[94]	[94]	
*Clostridium butyricum*		[80]			
*Clostridium clostridioforme*		[70]	[70]	[70]	[70]
*Clostridium cochlearium*	[76]				
*Clostridium difficile*		[29,48,76]	[29,76]	[29,76,92]	
*Clostridium ghoni*					[76]
*Clostridium lentoputrescens*	[76]			[92]	
*Clostridium limosum*	[76]				
*Clostridium lituseburense*			[76]	[76]	
*Clostridium malenomenatum*	[76]				
*Clostridium mangenoti*					[76]
*Clostridium paraperfringens*				[92]	
*Clostridium paraputrificum*		[29,80]			
*Clostridium perfringens*		[29,70]		[92]	[29]
*Clostridium propionicum*			[76]	[76]	
*Clostridium putrefaciens*			[76]	[76]	
*Clostridium saccharolyticum*		[93]	[93]	[93]	
*Clostridium septicum*		[29,80]			
*Clostridium sordellii*				[76,92]	[76]
*Clostridium sporogenes*		[80]		[95]	[95]
*Clostridium sticklandii*			[76,94]	[76,94]	
*Clostridium subterminale*			[76]	[76]	
*Clostridium tetani*	[76]				
*Clostridium tetanomorphum*	[41,76]				
*Faecalibacterium prausnitzii*		[93]	[93]	[93]	[93]
*Peptostreptococcus anaerobius*				[95]	
*Peptostreptococcus asaccharolyticus*	[29]				
Enterococcaceae					
*Enterococcus faecalis*	[80] ^a^				[96]
*Enterococcus faecium*					[97]
*Eubacteriaceae*					
*Eubacterium cylindroides*			[93]	[93]	[93]
*Eubacterium hallii*			[93]	[93]	
*Eubacterium rectale*		[93]	[93]	[93]	[93]
Lactobacillaceae					
*Lactobacillus acidophilus*					[97,98]
*Lactobacillus alimentarius*					[97]
*Lactobacillus brevis*					[97]
*Lactobacillus casei*			[46]	[46]	[76]
*Lactobacillus casei Shirota*					[70]
*Lactobacillus confusus*					[97] ^b^
*Lactobacillus coryniformis*					[99]
*Lactobacillus fermentum*					[97]
*Lactobacillus helveticus*			[46]	[46]	[46]
*Lactobacillus hilgardii*					[97]
*Lactobacillus johnsonii*					[98]
*Lactobacillus pentosus*					[100]
*Lactobacillus plantarum*					[97,100,101,102]
*Lactobacillus rhamnosus*					[97,98]
*Lactobacillus sanfranciscencis*					[97,101]
Lachnospiraceae					
*Anaerostipes caccae*		[93]	[93]	[93]	
*Anaerostipes hadrus*		[93]	[93]	[93]	
*Butyrivibrio fibrisolvens*		[93]	[93]	[93]	
*Roseburia intestinalis*		[93]	[93]	[93]	[93]
*Roseburia inulinovorans*		[93]	[93]	[93]	
*Ruminococcaceae*					
*Ruminococcus obeum*		[93]	[93]	[93]	[93]
*Ruminococcus sp.*		[93]	[93]	[93]	
*Ruminooccus torques*		[93]	[93]	[93]	
*Staphylococcaceae*					
*Staphylococcus epidermidis*	[80] ^c^	[80] ^c^			
Veillonellaceae					
*Megamonas hypermegale*		[93]	[93]	[93]	[93]
**BACTEROIDETES**					
Bacteroidaceae					
*Bacteroides distasonis*				[92]	
*Bacteroides eggerthii*			[93]	[93]	
*Bacteroides fragilis*	[29]	[29,80,93]	[92,93]	[29,70,92,93]	
*Bacteroides gingivalis*				[92]	
*Bacteroides ovatus*			[29,93]	[29,92,93]	[29]
*Bacteroidesruminicola* subsp. *brevis*				[92]	
subsp. *Ruminicola*				[92]	
*Bacteroides thetaiotaomicron*		[29,70]	[70,93]	[29,70,92,93]	
*Bacteroides uniformis*		[93]	[93]	[93]	
*Bacteroides vulgatus*			[93]	[93]	
*Porphyromonadaceae*					
*Parabacteroides distasonis*		[93]	[93]	[93]	
**ACTINOBACTERIA**					
Bifidobacteriaceae					
*Bifidobacterium adolescentis*		[29,93]	[29,93]	[93]	
*Bifidobacteriumanimalis* subsp. *Lactis*			[70]		[70]
*Bifidobacterium bifidum*		[29]	[29]		
*Bifidobacterium infantis*		[29,93]	[29,93]	[93]	[93]
*Bifidobacterium longum*	[29]		[29]		[29,70]
*Bifidobacterium pseudolongum*		[29]	[29]		
*Bifidobacterium sp.*		[80]			
**PROTEOBACTERIA**					
Enterobacteriaceae					
*Citrobacter freundii*	[40] ^d^, [42]				
*Citrobacter intermedius*	[40] ^e^				
*Enterobacter aerogenes*	[40] ^f^				
*Escherichia coli*	[29,40,80]				
*Morganella morganii*	[40] ^g^				
*Proteus sp.*	[80]				
**FUSOBACTERIA**					
Fusobacteriaceaea					
*Fusobacterium* sp*.*		[80]			

^a^ mentioned in original paper as *Streptococcus faecalis*; ^b^ mentioned in original paper as *Weisella confusa*; ^c^ mentioned in original paper as *Staphylococcus albus*; ^d^ mentioned in original paper as *Escherichia freundii*; ^e^ mentioned in original paper as *Escherichia intermedia*; ^f^ mentioned in original paper as *Aerobacter aerogenes*; ^g^mentioned in original paper as *Proteus morganii*.

**Table 2:** Studies describing associations between *p*-cresyl sulfate concentrations and clinical parameters and outcomes of patients with chronic kidney disease.

Patient Type	Patient Number	Total or Free pCS Concentration	Association	Ref.
Diabetic nephropathy	209	total	CAD	[118]
Stable angina	202	total	Severity of CAD	[126]
Stable angina with early CKD	154	total	QTc prolongation	[125]
CKD and stable angina	403	total	LV systolic function	[122]
CKD	72	total	CV and dialysis event (progression)	[121]
	149	free and total free	IL-6 and PWV Plasma glutathione peroxidase	[123]
	200	urinary excretion	CV event	[129]
	268	total	Renal progression and all-cause mortality	[131]
	320	total	Pruritus	[132]
CKD and CAD	340	total	MACE	[127]
CKD and HD (32%)	139	free	Survival	[133]
HD	91	free	Survival and function of vascular access	[117]
	100	total	Ankle Brachial index, AV-shunt failure and vascular access failure event	[120]
	209	total	Co-morbidity of CAD and DM *	[119]
	394	total	CV mortality and first CV event	[124]
Elderly HD	112	free	All-cause and CV mortality	[128]

AV: arterio-venous; CAD: coronary artery disease; CKD: chronic kidney disease; CV: cardiovascular; DM: diabetes mellitus; HD: hemodialysis; IL-6: interleukin-6; LV: left ventricle; MACE: major cardiovascular events; pCS: *p*-cresyl sulfate; PWV: pulse wave velocity; QTc: heart-rate corrected QT interval; * not with pro-inflammatory markers.

**Table 3:** Targets and methods to reduce *p*-cresyl sulfate concentrations.

Target	Method
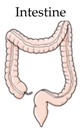	1. Affecting the generation of pCS
	*Diet*:
	↑ Carbohydrate/fiber (risk for hyperphosphatemia, hyperkalemia in CKD) versus ↓ Protein intake (risk for malnutrition in HD)
	*Pro-*, *pre-*, *synbiotics*:
	↑ Saccharolytic activity of bacteria
	*Laxatives*
	↓ Colonic transit time
	2. Adsorption
	Oral sorbent AST-120
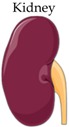	3. Preserving kidney function
	Preserving *tubular secretion* (transporter expression)
	4. Renal replacement therapy
	*Dialysis*: combining convective and diffusive removal *Renal transplantation*
